# Label-Free and Highly-Sensitive Detection of Ochratoxin A Using One-Pot Synthesized Reduced Graphene Oxide/Gold Nanoparticles-Based Impedimetric Aptasensor

**DOI:** 10.3390/bios11030087

**Published:** 2021-03-19

**Authors:** Yasmin Alhamoud, Yingying Li, Haibo Zhou, Ragwa Al-Wazer, Yiying Gong, Shuai Zhi, Danting Yang

**Affiliations:** 1 Zhejiang Key Laboratory of Pathophysiology, Department of Preventative Medicine, School of Medicine, Ningbo University, 818 Fenghua Road, Ningbo 315211, China; yasminalhamoud@zju.edu.cn (Y.A.); 176001260@nbu.edu.cn (Y.L.); 176001002@nbu.edu.cn (Y.G.); 2 Institute of Pharmaceutical Analysis and Guangdong Province Key Laboratory of Pharmacodynamic Constituents of Traditional Chinese Medicine & New Drug Research, College of Pharmacy, Jinan University, Guangzhou 510632, China; haibo.zhou@jnu.edu.cn; 3 Department of Pharmacy, Faculty of Applied Medical Sciences, Yemeni Jordanian University, 1833 Sana’a, Yemen; a_ragwa@outlook.com

**Keywords:** Ochratoxin A, electrochemical biosensor, gold nanoparticles, three-dimensional reduced graphene oxide, aptamer

## Abstract

Ochratoxin A (OTA) primarily obtained by the genera *aspergillus* and *penicillium*, is one of the toxic substances for different organs and systems of the human body such as the kidney, liver, neurons and the immune system. Moreover, it is considered to cause tumors and fetal malformation even at a very low concentration. Fast and sensitive assay for detection of OTA at ultralow levels in foods and agricultural products has been an increasing demand. In this study, a new label-free electrochemical biosensor based on three-dimensional reduced graphene oxide/gold nanoparticles/aptamer for OTA detection was constructed. The 3D-rGO/Au NPs nanocomposites were firstly synthesized using a one-pot hydrothermal process under optimized experimental conditions. The 3D-rGO/Au NPs with considerable particular surface area and outstanding electrical conductivity was then coated on a glass carbon electrode to provide tremendous binding sites for -SH modified aptamer via the distinctive Au–S linkage. The presence of OTA was specifically captured by aptamer and resulted in electrochemical impedance spectroscopy (EIS) signal response accordingly. The constructed impedimetric aptasensor obtained a broad linear response from 1 pg/mL to 10 ng/mL with an LOD of 0.34 pg/mL toward OTA detection, highlighting the excellent sensitivity. Satisfactory reproducibility was also achieved with the relative standard deviation (RSD) of 1.393%. Moreover, the proposed aptasensor obtained a good recovery of OTA detection in red wine samples within the range of 93.14 to 112.75% along with a low LOD of 0.023 ng/mL, indicating its applicability for OTA detection in real samples along with economical, specific, susceptible, fast, easy, and transportable merits.

## 1. Introduction

Ochratoxin A is one of the most popular mycotoxins that is produced by two genera of fungi: *Penicillium* and *Aspergillus*, which can be found in many types of food consumed daily such as cereals, coffee, wine, spices, and dairy products [1,2,3]. Ochratoxin A (OTA) is found to cause severe harmful effects including genotoxic, neurotoxic, carcinogenic, and nephrotoxic effects on both animals and humans [4]. Accordingly, there is an urgent necessity to find accurate, selective, specific, and highly sensitive methods for detecting OTA even if it is found in very low concentrations. To this day, there are many accurate and highly efficient traditional methods such as chromatography (HPLC, LC/MS) [5,6] and enzyme-linked immunosorbent assay (ELISA) [7] that have been widely used to detect OTA in food, but the limitations of these methods are that they require highly skilled personnel, advanced equipment and time-consuming preparation and detection. Recently, electrochemical biosensors have attracted great attention by researchers as a reliable and fast point-of-care application in various fields. Furthermore, the occurrence of aptamers as the remarkable recognition probes with high specificity and affinity, fast response, simple operation, low cost, and miniaturization, have been used as convenient, sensitive, and robust methods for OTA monitoring with the necessary help of advanced nanomaterials in foodstuffs [8,9]. The electrochemical aptasensors are classified into label-free and label-based types relying on whether the detection signals are from redox-labelled aptamers [10] or the redox probe in the electrolyte [11]. The redox-labelled aptamer EC sensor exhibits high sensitivity; however, the complex construction and high cost limited its development [12]. Thus, the label-free EC aptasensor based on a redox probe such as [Fe (CN)_6_]^3−/4−^ or MB in the electrolyte was developed to overcome the above problem [13]. However, low stability of aptamer immobilized on the electrode and low sensitivity of detection signals are still critical problems in label-free based aptasensor construction. 

Graphene has been widely used in the fabrication of electrochemical biosensors due to its outstanding physical properties and electrical conductivity [14]. However, the surface development is still intrinsically restricted by the two-dimensional (2D) nature which suffers from the poor processability and the agglomeration or re-stacking of graphene nanosheets that diminishes its intrinsic properties (electron transport and restricts application) [15]. In order to avoid the re-stacking of individual sheets, some endeavors have been made recently to develop three-dimensional (3D) structure graphene [16,17]. These unique interconnected network structures of 3D graphene improve the properties and features of 2D graphene, giving it high electrical conductivity, large surface area, high porosity, and distinct mechanical merits [18,19]. Reduced graphene oxide (rGO), allowing more immobilized nanoparticles to be deposited on its large surface area with high stability, has been preferred in the electrochemical aptasensors construction over graphene [20,21]. Moreover, gold nanoparticles (AuNPs) can be embedded in rGO to construct aptasensor with a highly charge transfer rate and considerable active surface area [22] along with promisingly bio-compatibility for anchoring thiolated/sulfhydryl DNA [23,24]. For instance, the AuNPs dotted rGO nanocomposites-based electrochemical aptasensors could achieve a high ultrasensitive detection of miRNA with a LOD of 1.73 pM [25]. In this regard, the 3D-rGO/Au NPs nanocomposites will be an excellent immobilization substrate for aptamer and high charge transporter for the development of ultrasensitive label-free based biosensor. 

There are two different ways to fabricate rGO-AuNPs nanocomposites, including ex situ hybridization and in situ decoration [26]. For instance, the rGO-AuNPs nanocomposites used for mycotoxin [27] or OTA detection [8,28] were synthesized via ex situ hybridization process, which means the AuNPs nanoparticles were prepared in advance and then adsorbed onto the surface of RGO through either covalent or noncovalent binding. In comparison with the ex situ hybridization process, the preparation of the in-situ synthesis approach is much easier [29], which could facilitate the fabrication process of the electrochemical electrode. Currently, there is no report of label-free based electrochemical aptasensor based on one-pot, in-situ decorated 3D-rGO/AuNPs nanocomposites for highly efficient and ultrasenstive detection of OTA in wine. In this work, we designed a novel impedimetric aptasensor using one-pot, in-situ synthesized 3D-rGO/Au NPs nanocomposites toward label-free and sensitive OTA determination. Parameters for 3D-rGO/Au NPs nanocomposites synthesis were optimized to provide high sensitivity and stability. The aptamer-3D-rGO/Au NPs/GCE system was formed via the Au-S covalent bond between the specific OTA aptamer and 3D-rGO-Au NPs/GCE substrate. When OTA is present, it will bind with aptamer, forming an OTA-aptamer curled complex on the surface of the modified electrode that causes the corresponding EC signal responses of [Fe (CN)_6_]^3−/4−^ redox probe. The proposed OTA aptasensor showed ultrasensitive detection limit, good reproducibility, high stability, and good recovery in real samples. 

## 2. Materials and Methods

### 2.1. Materials and Chemicals

Methanol and potassium chloride (KCl) were purchased from the Aladdin company (Shanghai, China), potassium ferrocyanide (K_4_[Fe(CN)_6_]), potassium ferricyanide (K_3_[Fe(CN)_6_]), disodium hydrogen phosphate dodecahydrate (Na_2_HPO_4_-12H_2_O), disodium hydrogen phosphate dehydrate (NaH_2_PO_4_-2H_2_O), sodium hydroxide (NaOH), and glucose (C_6_H_12_O_6_), were purchased from Sinopharm Chemical Reagent Co., Ltd. (Shanghai, China). Chloroauric acid (HAuCl_4_·4H_2_O), graphene oxide nanosheets (GO, XF002-1, 500 nm–5 μm; ~99%, Hummers) were purchased from Nanjing XFNANO Materials Tech. Co., Ltd (Nanjing, China). Tris(2-carboxyethyl) phosphine (TCEP) was obtained from Sigma-Aldric. Ochratoxin A (OTA), ochratoxin B (OTB), deoxynivalenol (DON), and zearalenone (ZEA) were purchased from Sigma-Aldrich (Shanghai, China). Tris(hydroxymethyl)aminomethane hydrochloride (tris-HCl) and ethylenediaminetetraacetic acid disodiumsalt (EDTA) were purchased from Sinopharm Chemical Reagent Co., Ltd. (Shanghai, China). Bovine serum albumin (BSA) was purchased from Sigma-Aldrich. OTA binding-aptamer with a sequence (5′-GAT CGG GTGTGG GTG GCG TAA AGG GAG CAT CGG ACA-(CH_2_)_6_-SH-3′) was purchased from Sangon Biotech. (Shanghai, China). All reagents and chemicals were of the highest analytical grade. Millipore-Q water (18.2 MΩ cm^−1^) was used to prepare all aqueous solutions.

### 2.2. Synthesis of 3D-rGO/Au NPs Nanocomposites

3D-rGO/Au NPs nanocomposites were fabricated through a one-pot hydrothermal reduction process using GO nanosheets, HAuCl_4_, and glucose. Key parameters for 3D-rGO/AuNPs such as amounts of glucose and HAuCl_4_·4H_2_O, were optimized to obtain well-dispersed and long-stabilized 3D-rGO/AuNPs. In brief, a 2 mg/mL suspension of GO was prepared by the sonication of GO nanosheets (20 mg) in water (10 mL) for 2 h. Then, the above dispersion was mixed with 10 mL glucose (2, 50, and 150 mg/mL) solution under sonication. The volume (200, 400, 1000, and 1600 µL) of HAuCl_4_·4H_2_O (2%, *w*/*w*) was introduced into the mixture and sonicated evenly for 1 h. Afterwards, the obtained homogenous solution was poured into a Teflon-lined autoclave and reacted at 180 °C for a full 12 h. After the autoclave was cooled to the room temperature, the aerogel was washed three times using distilled water and blotted with filter paper to remove surface-adsorbed water. At last, the obtained 3D-rGO/Au NPs hydrogel was freeze-dried (−50 °C) for 48 h to get the dry 3D-rGO/Au NPs nanocomposites. For comparison of rGO and rGO/AuNPs, rGO was synthesized with the same protocol of rGO/AuNPs without the addition of HAuCl_4_·4H_2_O. In addition, the role of glucose and high temperature in the thermal reduction was investigated. 

### 2.3. Preparation of the Aptasensor Surface

Firstly, the glassy carbon electrode (GCE) was polished with alumina powder (diameters 1.0 and 0.05 μm) until a mirror-like surface was obtained and then was sonicated in water and absolute ethanol consequently. After the cleaned electrode was dried at room temperature, 8.0 μL of 3D-rGO-AuNPs nanocomposite suspension was drop-casted onto the surface of GCE and left to dry for about 2 h at room temperature. Subsequently, the aptamer was activated by 100 mM TCEP and incubated for 1 h at 37 °C. Then 10 μL of activated aptamer was dropped on the surface of the modified electrode. After incubated for 2 h at room temperature, the unbound aptamers were excluded from the modified electrode surface by careful washing with phosphate buffered saline (PBS) solution three times. After drying, the obtained aptasensor was stored at 4 °C for the following experiments. 

### 2.4. Electrochemical Measurement 

In order to monitor the changes occurring effectively and comprehensively on the modified electrode surface both electrochemical impedance spectroscopy (EIS) and cyclic voltammetry (CV) were used. EIS was recorded in 0.1 M PBS buffer with pH 7.4 in the presence of 1 mM [Fe (CN)_6_]^3−/4−^ and 0.1 M KCl at a bias potential of +0.20 V, an amplitude of 5 mV and a frequency range from 0.1 to 10^6^ Hz. CV measurements of various fabricated electrodes were performed with potential from −0.6 to +0.6V and scan rate of 100 mV/s. For OTA detection, the prepared aptasensor was dipped in a 0.1 M PBS buffer (pH 7.4) containing OTA for 1 h. Then OTA-captured electrodes were rinsed with PBS buffer three times. After drying at room temperature, the EIS measurements were performed to detect OTA.

### 2.5. Instrumentation

All the electrochemical measurements were conducted using a model CHI 660D electrochemical workstation (Shanghai Chenhua Instruments Co. Ltd., Shanghai, China). A 3-mm-diameter GCE was used as the working electrode, a platinum electrode as the auxiliary electrode, and an Ag/AgCl saturated with KCl as the reference electrode are the system elements that composed the three-electrode system for electrochemical experiments. All the potentials were reported with respect to the reference electrode. The structure and morphology of synthesized nanocomposites was checked by scanning electron microscopy (SEM) (S-3000N, Hitachi, Tokyo, Japan) and transmission electron microscopy (TEM) (H-7650, Hitachi, Tokyo, Japan). Energy-dispersive X-ray spectroscopy (EDS) elementary analysis was performed with the VEGA-3-SBH (Tescan, Brno, Czech). X-ray diffraction (XRD) patterns were carried out with the D8 Advance X-ray powder diffractometer (Bruker, Berlin, Germany). Raman spectroscopy was recorded by a Raman microscope with a 532-laser source from 800 to 3600 cm^−1^, with an exposure time of 1 s and an accumulation number of 10. UV-vis spectra were obained with a D8 Spectrometer (Feile Instruments, Nanjing, China). Drying of 3D-rGO/AuNPs nanocomposites under vacuum was done with a Freeze dryer (Ningbo Shuangjia Instruments, Ningbo, China). The pH measurements were done with a Metrohm model 691 pH/mV meter (Metrohm, Herisau, Switzerland).

## 3. Results

### 3.1. Principles of 3D-rGO/Au NPs Nanocomposites Based Label-Free Aptasensor for OTA

**Figure 1:**
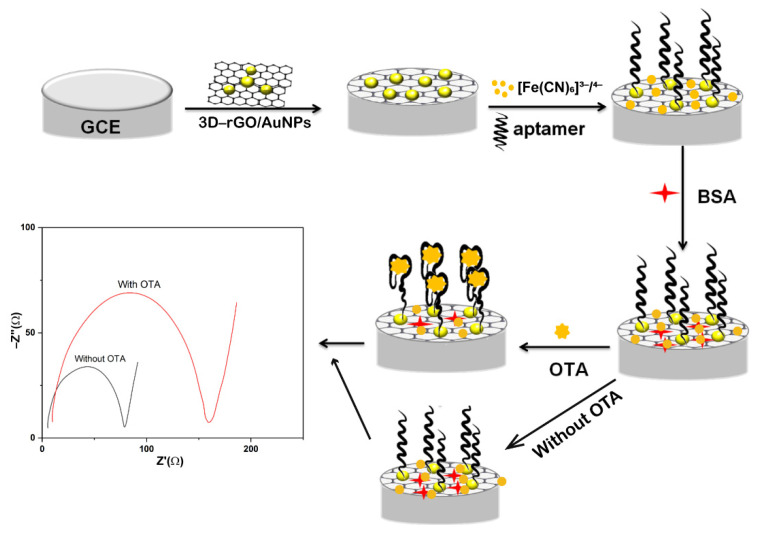
Schematic illustration of GCE/3D-rGO/AuNPs based aptasensor for Ochratoxin A (OTA).

As described in Figure 1, a label-free and highly sensitive impedimetric aptasensor for detection of OTA based on 3D-rGO/AuNPs nanocomposites was designed. 1 mM [Fe (CN)_6_]^3−/4−^ with 0.1 M KCl in the PBS solution was used as the redox probe to record the EIS response of every step. Firstly, the surface of GCE was modified by drop-coating nanocomposites of 3D-rGO-AuNPs. The value of the charge transfer resistance decreased compared with the bare GCE due to the great conducting ability and the increased electron transfer rate of rGO and AuNPs. The resultant film was then bound with aptamer via Au-S covalent bonds and formed the GCE-3D-rGO-AuNPs-aptamer system. Here, the electron transfer is hampered by its isolating effect, leading to the increase of the charge-transfer resistance of the electrode. Then the modified electrode was incubated with 1.0% BSA to block any possible non-specific binding as commonly used in biosensing [30,31]. When OTA-aptamer was specifically combined on the aptasensing surface, this obtained the curled complex of OTA-aptamer which partly blocked the electron transfer path and then the EIS signal responses changed with the according OTA concentration. 

### 3.2. Optimization and Characterization of 3D-rGO/AuNPs Nanocomposites

Glucose with abundant oxidative groups and nontoxic merit, was shown to be an excellent reducing agent to reduce GO into rGO in the hydrothermal process [32,33] and to reduce Au^3+^ to Au [29] as well. As shown in Figure S1, the sample “b” was synthesized without high temperature in the presence of glucose (detailed parameters can be seen in Table S1) and exhibited the yellow-brown color of GO and a much lower CV current peak than that of sample “c” (Figure S2D); this indicated that sample b was not reduced compared to the dark color of our proposed sample “c” at high temperature. Furthermore, with the fabrication of sample “b” on the GCE, the Rct was larger than the bare GCE (Figure S2A), indicating the insulating property of sample “b”. It can be concluded that glucose can only perform its reduction role under high temperature to synthesize rGO/AuNPs. In addition, under high temperature, GO could be reduced to rGO despite the absence of glucose and enabled the analysis of OTA through layer-by-layer fabrication as seen in Figure S2C. However, the maximum current peak of the CV curves of sample “c”, synthesized with the presence of glucose, is 1.3~fold higher than that of sample “d” (Figure S2D). Moreover, the absence of glucose could cause the rGO sentiment in 5 min as shown in sample “d”, compared with our proposed stable sample “c” in one month. In comparison of ΔR_CT_ between BSA and OTA fabrication on GCE (Figure S2B,C), it was found that the ΔR_CT_ (OTA-BSA) of sample “c” (52 ± 5 Ω) is larger than that of sample “d” (34 ± 10 Ω) with smaller relative standard deviation (RSD), which indicates the glucose performs as the stabilizer in the synthesis of rGO/AuNPs in addition to reducing agent [29].

**Figure 2:**
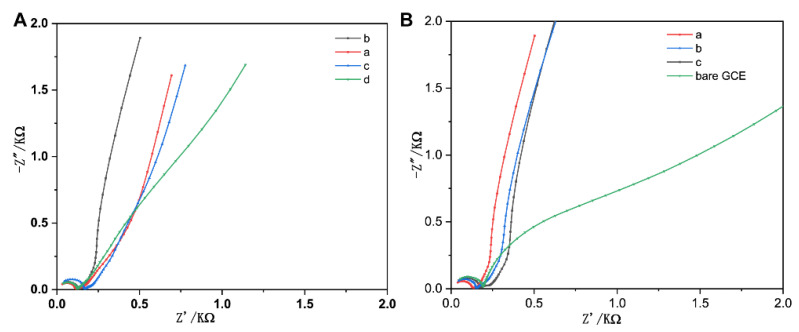
Electrochemical impedance spectroscopy (EIS) measurements of (**A**) different volumes of HAuCl_4_·4H_2_O (a, 200 µL; b, 400 µL; c, 1000 µL; d, 1500 µL) and (**B**) different amounts of glucose (a, 20 mg; b, 500 mg; c, 1000 mg) for 3D-rGO/AuNPs nanocomposites and bare glassy carbon electrode (GCE).

As the critical role of gold amounts and glucose in the system, the amounts of HAuCl_4_ (200, 400, 1000, and 1600 µL) (Table S2) and glucose (20, 500, and 1500 mg) (Table S3) were firstly optimized to obtain the largest charge-transfer resistance change and the fastest ion diffusion ability, respectively. EIS was used to investigate the characteristics of charge and ion transfer in the 3D-rGO/AuNPs at different synthesized conditions in a frequency range of 10^6^ Hz to 0.1 Hz. The diameter of the semicircle in the high-frequency range gives an approximate value of the charge–transfer resistance (Rct) at the electrode/electrolyte interface and the straight slope line in the low-frequency region is ascribed to the diffusive resistance of the electrolyte in the electrode pores and proton diffusion in the host materials. It can be seen in Figure 2A, sample “b” showed a more vertical straight line than samples “a, c, and d”, indicating the faster ion diffusion ability of the porous rGO materials. We can also see from the Figure 2B, the sample “a” exhibited the smallest semicircle among sample “a, b, c and bare GCE”, suggesting the loading of sample “a” onto the GCE surface resulted in the highest decrease in charge–transfer resistance to bare GCE. Thus, the sample exhibiting the most vertical straight line along with the smallest semicircle was chosen as the optimized nanomaterial, which means the synthesized condition was set as 400 µL of HAuCl_4_, 20 mg glucose, and 20 mg GO in 20 mL aqueous solution. 

#### 3.2.1. Morphology and Structure Characterization of 3D-rGO/AuNPs Nanocomposites

**Figure 3:**
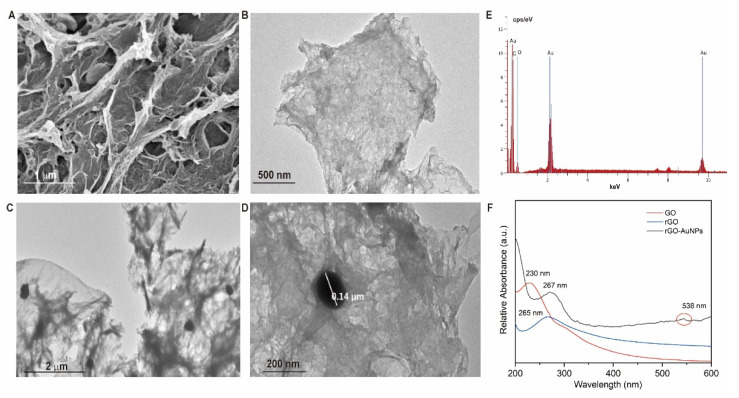
(**A**,**B**) Scanning electron microscopy (SEM) images of the prepared of 3D-rGO/AuNPs at different magnifications; (**C**,**D**) transmission electron microscopy (TEM) images of reduced graphene oxide (RGO) and rGO-AuNPs; (**E**) energy-dispersive X-ray spectroscopy (EDS) of 3D-rGO/AuNPs; and (**F**) UV-vis spectra of graphene oxide (GO), reduced graphene oxide (rGO) and rGO-AuNPs nanocomposites.

SEM and TEM have been used to characterize the morphology and structure of synthesized nanomaterials. The SEM and TEM images of 3D-rGO/AuNPs nanomaterials are illustrated in Figure 3A,C,D, respectively. As shown in Figure 3A, the nanocomposite has high porosity and a sponge-like structure with AuNPs clearly embedded in it. Compared with the TEM image of rGO in Figure 3B, the distribution of AuNPs (black dots, size of ~140 nm shown in Figure 3D) in the porous structure of rGO is clearly seen in Figure 3C, which is important and necessary for chemisorption of aptamer during the immobilization step via Au-S linkage. According to the EDS elemental analysis (Figure 3E), the presence of Au, C, O in our nanomaterial was evidenced along with the percentage of Au NPs content of 1.74%. UV−vis absorption spectrometry was also used to identify the presence of rGO and AuNPs. As was shown in Figure 3F the pure GO exhibited a typical absorption peak at 230 nm. After the hydrothermal process, the 230 nm peak was shifted to 265 nm, indicating the change from GO to rGO, which could be seen in both rGO and rGO/AuNPs. The second peak at 538 nm was assigned to Au NPs. This result accords to the previous report [29,34], illustrating the successful formation of rGO/AuNPs.

**Figure 4:**
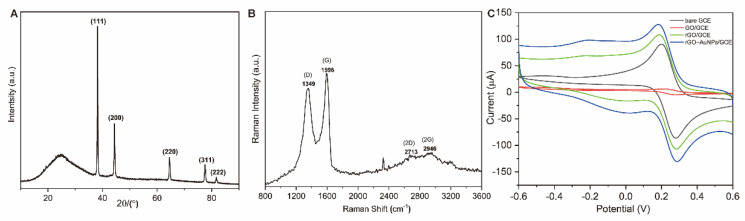
(**A**) X-ray diffraction (XRD) patterns of 3D-rGO/AuNPs nanocomposites; (**B**) Raman spectrum of 3D-rGO/AuNPs nanocomposites; (**C**) cyclic voltammetry (CV) curves of bare GCE, GO/GCE, 3D-rGO/GCE, and 3D-rGO-AuNPs/GCE.

For further investigation, XRD was applied to study the purity and crystallinity of 3D-rGO-AuNPs nanomaterials. Figure 4A demonstrates the wide angle XRD profiles of 3D-rGO/AuNPs. As we can see, the pattern of the sample contains broad reflections at 2θ = ~20 and 30°, which means that non-defect and high crystalline graphene was synthesized [21], indicating the formation of the 3D-rGO structure during the hydrothermal process. The five well-resolved diffraction peaks at 2θ value of ~38, 44, 64, 77, and 81° can be indexed as monoclinic Au 111, 200, 220, 311, and 222 reflections, exhibiting the formation and presence of AuNPs. The Raman technique is one of the most efficient and nondestructive methods that enables investigating the quality and structure of carbon compounds. The Raman spectrum of 3D-rGO-AuNPs nanocomposite is shown in Figure 4B, exhibiting two prominent peaks at 1349 and 1600 cm^−1^, which correspond to the D and G bands of rGO, respectively. The D band indicates the presence of defects caused by the sp^3^ hybridized carbon atoms, while the G band is a typical peak exhibited by the pristine sp^2^ lattice carbon atoms in the graphene sheet. The broadened 2D peak at 2700 cm^−1^ indicates that GO was reduced and composed of multi-layers of stacked graphene nanosheet [35,36,37,38]. In addition, the anodic peak current of bare GCE was reduced from 76.2 μA to about 0.9 μA, which was due to the insulating properties of non-reduced GO layer on GCE resulting in the repulsion of [Fe(CN)_6_]^3−/4−^ on the GCE surface^−^. However, when GCE was modified with rGO, [Fe(CN)_6_]^3−/4−^ could diffuse through the porous structure of reduced nanomaterial and improve the redox activity. The electro-conductivity of GCE was further improved with the 3D-rGO/AuNPs nanomaterial as seen by the highest peak current of 128 μA in Figure 4C, attributing to the synergic conductive performance of rGO and AuNPs which is in accordance with results in the published articles [29,39].

#### 3.2.2. Electrochemical Characterization of the Aptasensor

(1) EIS measurements

**Figure 5:**
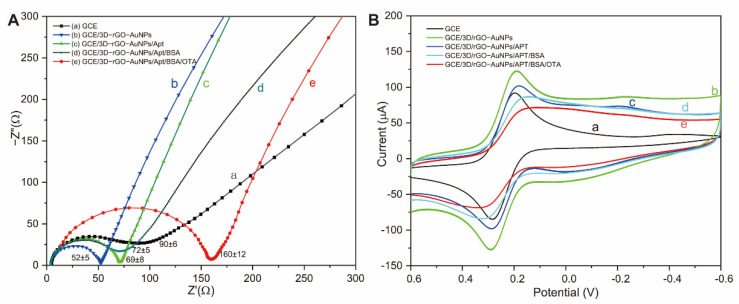
(**A**) Nyquist plots and (**B**) CV curves of (**a**) the bare GCE, (**b**) GCE/3D-rGO-AuNPs, (**c**) GCE/3D-rGO-AuNPs/aptamer, (**d**) GCE/3D-rGO-AuNPs/aptamer/BSA, and (**e**) GCE/3D-rGO-AuNPs/aptamer/BSA/OTA in a solution of phosphate buffered saline (PBS) pH 7.4 containing 1 mmol L^−1^ [Fe (CN) _6_]^3−/4−^ and 0.1 mol L^−1^ KCl.

EIS is one of the effective methods applied to monitor the changes occurring on the modified electrode surface during various modification steps and thus verifying the success of the modification or immobilization processes. Nyquist plots were chosen to represent the EIS measurements. As shown in Figure 5A, lines “a–e” show the different modification levels which was performed on the electrode surface. As evident, the semicircle diameter of line “a” is larger than that of line “b”, suggesting that the loading of 3D-rGO-AuNPs onto GCE results in a decrease of charge–transfer resistance. R_CT_ value dramatically increased (shown as line “c”) after the aptamer was captured on the 3D-rGO-AuNPs-modified electrode surface, which could be interpreted as a result of the repulsion between the negatively charged [Fe(CN)_6_]^3−/4−^ present in the standard solution and the negatively charged phosphate present in the aptamer structure. The R_CT_ value of Line “d” slightly increased from 69 ± 8 to 72 ± 5 after incubation with BSA for non-specific blocking, which indicating BSA will not affect the sensitivity of the system. Finally, after the OTA presented, the R_CT_ value increased significantly (Figure 5A (e)), which refers to the fact that OTA was captured by its aptamer, thus blocking the reaction sites on the sensing surface.

(2) Cyclic voltammetry (CV)

CV measurements, another efficient method for monitoring the interface properties of the sensor surface, were also taken. Figure 5B shows the CV curves of the modified electrodes at different stages. After the 3D-rGO/AuNPs suspension was deposited on the bare electrode surface, the CV peak current obviously increased (Figure 5B), which indicates the coverage of electrode surface with a layer of 3D-rGO/AuNPs nanocomposite. The modified film facilitates the electrons transfer of the [Fe (CN)_6_]^3−/4−^ pair and enables a larger amount of loaded aptamer by providing a greater specific surface area. When aptamer was incubated with the above modified electrode, the peak current patently decreased due to the role of the aptamer acting as an isolating barrier to electron transfer. (Figure 5B (c)), illustrates the successful immobilization of aptamer on the electrode. After incubation of BSA, a small decrease is shown, indicating the successful fabrication of blocking BSA (Figure 5B (d)). An additional significant decrease in the peak current value occurred due to increased electron transfer resistance after the incubation with OTA, (Figure 5B (e)), suggesting that the proposed OTA aptasensor was successfully achieved.

### 3.3. Optimization of Aptasensing Parameters

To obtain the best analytical performance of the proposed strategy, the most important testing variables such as 3D-rGO/AuNPs concentration, aptamer concentration, aptamer incubation time on the electrode, and aptamer-OTA incubation time were optimized, respectively. EIS is very sensitive to changes in interfacial impedance occurring at the surface/electrolyte interface based upon biorecognition events [40]. Thus, the difference between the values of the charge–transfer resistance (ΔR_CT_) of GCE after BSA and OTA incubation was used for the evaluation of aptasensor analytical performance. The concentration of OTA was set as 1 ng·mL^−1^ for the optimization. 

The concentration of 3D-rGO/AuNPs nanocomposites aqueous suspension fabricated on the GCE can influence processes. The effect of different concentrations (0.1, 0.3, 0.5, 1, and 2 mg/mL) of the nanocomposites on the response of the electrochemical aptasensor was investigated. It is clear in Figure S3A that the 3D-rGO/AuNPs suspension concentration of 0.5 mg/mL exhibits the highest value of the charge–transfer resistance (R_CT_). However, EIS from samples of 1 and 2 mg/mL were not obtained; the modified film of those fell into the solution before completing the examination. The high concentration of 3D-rGO/AuNPs nanocomposites may cause a thick and heavy film on the electrode surface, which leads to weak fixation for a long time needed by EIS operation. In contrast, the EIS response of 0.5 mg/mL 3D-rGO/AuNPs nanocomposites was satisfied based on the difference between the values of the charge–transfer resistance (R_CT_) of aptamer and OTA. Therefore, 0.5 mg/mL was selected as the optimum concentration of 3D-rGO/AuNPs suspension for the following experiments. 

The concentration of aptamer is one of the analytical variables that may affect the aptasensor response significantly. Therefore, we compared different concentrations of aptamer (0.1, 0.5, 0.8, 1, and 2 µM) during the fabrication process. Figure S3B shows the effects of different-concentrations of aptamer on the electrochemical signal of the aptasensor by using 1 ng/mL OTA as the target. The EIS response was gradually increased from 0.1 to 1 µM owing to the filling out of whole active sites of the 3D-rGO/AuNPs modified electrode. The saturation level of the modified electrode surface was inferred by adopting the aptamer concentration of 1 µM, which is selected as the optimized parameter for the following experiments.

The time of incubating aptamer with the electrode surface was optimized as well. As seen in Figure S3C, the R_CT_ value magnified with increasing time and achieved a maximum value at 180 min. Consequently, 180 min was adopted as the optimum aptamer incubation time. The incubation time of the OTA with its aptamer is also one of the most important parameters that impacts the analytical condition of the presented aptasensor. Finally, different OTA-aptamer incubation time from 10 to 90 min were examined, respectively. As shown in the Fig S3D, the R_CT_ value increased notably with the increasing incubation time of OTA and reached the highest value (saturation level) at 60 min. The R_CT_ value decreased when the incubation time of OTA further increased. In order to get an aptasensor with fast response to OTA, 60 min was confirmed as the best incubation time. 

Thus, 0.5 mg/mL of 3D-rGO/AuNPs suspension, 1 µM of aptamer, 180 min of incubating aptamer with the electrode, and 60 min of incubating OTA with its aptamer were chosen as the optimal parameters. 

### 3.4. Analytical Performance of Electrochemical Aptasensors for OTA

**Figure 6:**
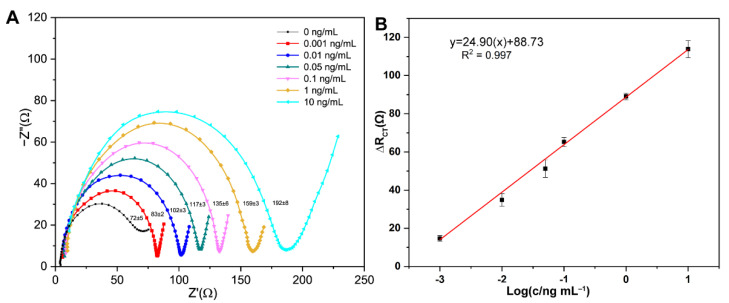
(**A**) Nyquist plots of aptasensor with different concentrations of OTA (0–10 ng/mL) and (**B**) The calibration curve of aptamer corresponding to the detection of OTA based on the change in electron-transfer resistance (R_CT_), which is presented as ΔR_CT_.

The analytical performance of the proposed electrochemical aptasensor for OTA detection of different concentrations (0, 0.001, 0.01, 0.05, 0.1, 1, and 10 ng/mL) under the optimum conditions were explored through EIS measurements. It is shown in Figure 6A, with the increasing concentration of OTA, the EIS responses were enhanced accordingly. By analyzing the results, we found that there is a good linear relationship between the value of ΔR_CT_ vs. the value of the logarithm of OTA concentration (log (c/ng·mL^−1^)), which is shown in Figure 6B. The linear correlation equation can be indicated as ΔR_CT_ = 88.73+ 24.90 log (c/ng·mL^−1^) (R^2^ = 0.997). The detection limit of OTA (S/N = 3) was 0.34 pg/mL. The present aptasensor in this study had a lower LOD, which indicated that this technique was preferable in the detection of OTA. (Comparison of the developed impedimetric aptasensor with those from previous reports are summarized in Table 1)

### 3.5. Selectivity and Reproducibility of the Electrochemical Aptasensors

**Figure 7:**
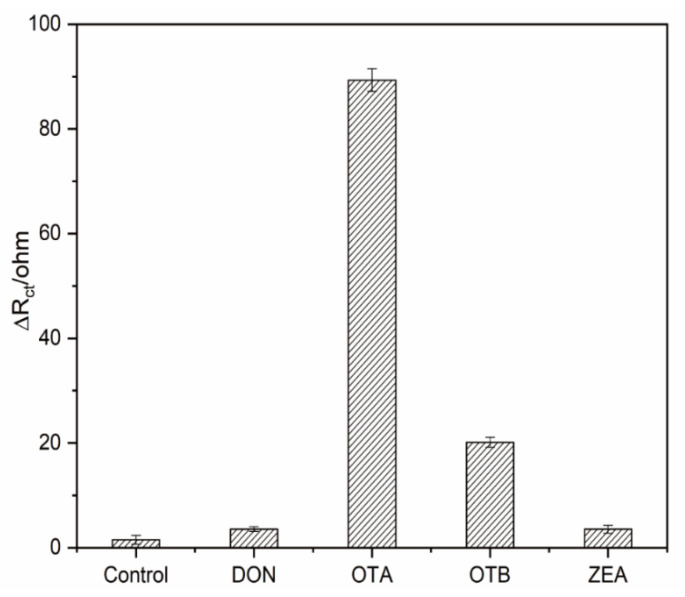
The specificity of the aptasensing system against OTA, ochratoxin B (OTB), deoxynivalenol (DON), and zearalenone (ZEA).

Selectivity and reproducibility are necessary factors to evaluate the efficiency of biosensors. Therefore, interferents OTB, DON, and ZEA were firstly employed to verify the selectivity of the designed aptasensor. The concentration for all the above interferents was set as 10 ng/mL while that for OTA was 1 ng/mL. As seen from Figure 7, the responses of DON and ZEA were almost the same as that of control test. Unfavorably, the proposed assay has a small cross-reactivity with OTB as also observed in other publications [45,46], which might be ascribed to their similar molecular structures and common antigen epitope. However, considering the response signal of OTA is ~4.5 fold of OTB with a 1/10 concentration, the selectivity of our system was acceptable. To check the reproducibility of the fabricated aptasensor, five replicates were performed under the same circumstances with responses of 160.15, 157.08, 159.59, 160.99 and 163.23 Ω, respectively. The relative standard deviation (RSD) was 1.393% for five measurements, indicating the high reproducibility of our sensor.

### 3.6. Real Sample Analysis

The practicability and reliability of the impedimetric aptasensor was demonstrated in real red wine. After a simple filtration process was performed to remove the large molecules using a 0.22 μm membrane, the filtered red wine samples were spiked with five different concentrations of OTA (0.05, 0.1, 0.5, 1, and 2 ng/mL). According to the above calibration curve, 0.05 ng/mL (ΔR_CT_ = 14.1 ± 0.96 Ω) was calculated as a much lower concentration of 0.001 ng/mL, which demonstrated the above calibration curve is not applicable to the red wine samples. It is assumed that the matrix effect from red wine decreased the response sensitivity of the aptasensor [47]. Thus, the new calibration curve for red wine (Figure S4) is established for better quantification. The linear correlation equation can be indicated as ΔR_CT_ = 39.09(x) + 64.89 log (c/ng·mL^−1^) (R^2^ = 0.998) with a LOD of 0.023 ng/mL, which was much lower than the European commission standard (2 ng/mL). The obtained recovery within the range of 93.14–112.75% was reasonable according to the investigation results given in Table 2, demonstrating good accuracy of developed aptasensor for OTA detection in real samples.

## 4. Conclusions

In summary, this study developed a novel 3D-rGO/AuNPs-based aptasensing device using easy modification steps on the bare GCE to obtain sensitive and selective determination of OTA. The large-size of AuNPs embedded 3D network structures of porous graphene aerogel can afford large surface (active) area, good electrical conductivity, and tremendous binding sites for aptamer immobilization. With the presence of OTA, EIS signals of the proposed aptasensor were increased due to the obstruction of electron transfer caused by the specific binding between aptamer and OTA. Under optimal conditions, the developed aptasensing showed excellent EC performance with a high linear response and broad range from 0.001 to 10 ng/mL with an ultrasensitive LOD of 0.34 pg/mL. The sensitive OTA aptasensor introduced in this work displayed rapidity, low cost, easy preparation, and good specificity with satisfied reproducibility, which could be potentially employed for detection of OTA in real samples as a portable and reliable tool. To be noted, the interference of OTB should be eliminated or reduced by developing on-column specific recognition of OTA with aptamer-based hybrid affinity monolithic column coupled with HPLC or LC for better application [48,49,50].

## Figures and Tables

**Table 1:** Comparison of the developed impedimetric aptasensors for OTA detection.

Transducer	Linear Range (ng·mL^−1^)	LOD (ng·mL^−1^)	Reference
Thionine and IrO_2_ NPs modified SPCE	0.004~40	5.6 × 10^−3^	[41]
Au-ATP-rGO composite modified Au Electrode	0.1~200	0.03	[28]
Self-supported np-Au microelectrode	0.01~5	5 × 10^−3^	[42]
Layer-by-layer self-assembly modified Au electrode	0.1~10.0	0.03	[43]
Silver metallization of aptamers on disposable screenprinted Au electrodes	0.001~100	7 × 10^−4^	[44]
3D-rGO/AuNPs modified GCE	0.001~10	3.4 × 10^−4^	This work

**Table 2:** Recoveries of OTA in red wine samples by the proposed aptasensor.

Sample	Spike (ng/mL)	Determined (ng/mL)	Recovery (%)
1	0.05	0.050 ± 0.002	100.4
2	0.1	0.099 ± 0.013	98.81
3	0.5	0.53 ± 0.053	105.55
4	1	0.93 ± 0.072	93.14
5	2	2.26 ± 0.253	112.75

## Supplementary Materials

The following are available online at https://www.mdpi.com/2079-6374/11/3/87/s1, Table S1: Investigations of glucose role in thermal reduction process for 3D-rGO/AuNPs nanocomposites, Table S2: Optimized parameters of different volumes of HAuCl_4_·4H_2_O for 3D-rGO/AuNPs nanocomposites, Table S3: Optimized parameters of different amounts of glucose for 3D-rGO/AuNPs nanocomposites; Figure S1: The optical images of sample a GO, b GO reduction without high temperature, c rGO/AuNPs with glucose and d rGO/AuNPs without glucose (detailed parameters were in Table S1), Figure S2: (A–C) The Nyquist plots of GCE after every step of fabrication of nanomaterial, aptamer, BSA, and OTA; (D) CV curves of sample b, c, and d fabricated GCE, Figure S3: Optimization of (A) concentrations of 3D-rGO/AuNPs, (B) concentrations of aptamer, (C) incubation time of aptamer, (D) incubation time of OTA with aptamer, Figure S4 Calibration curve of OTA detection in red wine based on ΔR_CT_ vs. log (OTA concentration).
