# Supplementary material for: Label-Free and Highly-Sensitive Detection of Ochratoxin A Using One-Pot Synthesized Reduced Graphene Oxide/Gold Nanoparticles-Based Impedimetric Aptasensor
Source: Biosensors (Basel). 2021 Mar 19;11(3):87. doi: 10.3390/bios11030087 (PMC8003581; doi:10.3390/bios11030087)
Supplement: Supplementary file 1 [file biosensors-11-00087-s001.pdf]

biosensors

Supplementary material

Label-Free and Highly-Sensitive Detection of Ochratoxin A Using One-Pot Synthesized Reduced Graphene Oxide/Gold Nanoparticles-Based Impedimetric Aptasensor

Yasmin Alhamoud [1], Yingying Li [1], Haibo Zhou [2], Ragwa Al-Wazer [3], Yiying Gong [1], Shuai Zhi [1,*] and Danting Yang [1,*]

[1] Zhejiang Key Laboratory of Pathophysiology, Department of Preventative Medicine, School of Medicine, Ningbo University, 818 Fenghua Road, Ningbo 315211, China; yasminalhamoud@zju.edu.cn (Y.A.); 176001260@nbu.edu.cn (Y.L.); 176001002@nbu.edu.cn (Y.G.)

[2] Institute of Pharmaceutical Analysis and Guangdong Province Key Laboratory of Pharmacodynamic Constituents of Traditional Chinese Medicine & New Drug Research, College of Pharmacy, Jinan University, Guangzhou 510632, China; haibo.zhou@jnu.edu.cn

[3] Department of Pharmacy, Faculty of Applied Medical Sciences, Yemeni Jordanian University, 1833 Sana'a, Yemen; a_ragwa@outlook.com

* Correspondence: zhishuai@nbu.edu.cn (S.Z.); yangdanting@nbu.edu.cn (D.Y.)

Table S1 Investigations of glucose role in thermal reduction process for 3D-rGO/AuNPs nanocomposites

Sample	GO (mg)	$HAuCl_4 \cdot 4H_2O$ (μL)	Glucose (mg)	Temperature	Reaction Time
a	20	0	0	RT	0
b	20	400	20	RT	12 h
c	20	400	20	180 ℃	12 h
d	20	400	0	180 ℃	12 h

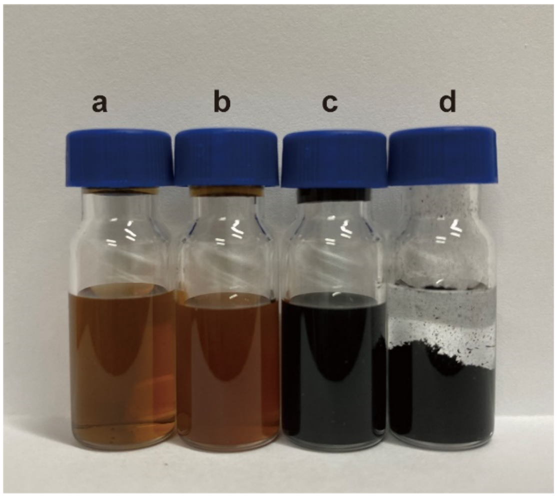

Figure S1 The optical images of sample a GO, b GO reduction without high temperature, c rGO/AuNPs with glucose and d rGO/AuNPs without glucose (detailed parameters were in Table S1)

Table S2 Optimized parameters of different volumes of $HAuCl_4 \cdot 4H_2O$ for 3D-rGO/AuNPs nanocomposites

Sample	GO (mg)	$HAuCl_4 \cdot 4H_2O$ (μL)	Glucose (mg)	Reaction time (h)
a	20	200	20	12
b	20	400	20	12
c	20	1000	20	12
d	20	1500	20	12

Table S3 Optimized parameters of different amounts of glucose for 3D-rGO/AuNPs nanocomposites

Sample	GO (mg)	$HAuCl_4 \cdot 4H_2O$ (μL)	Glucose (mg)	Reaction time (h)
a	20	400	20	12
b	20	400	500	12
c	20	400	1500	12

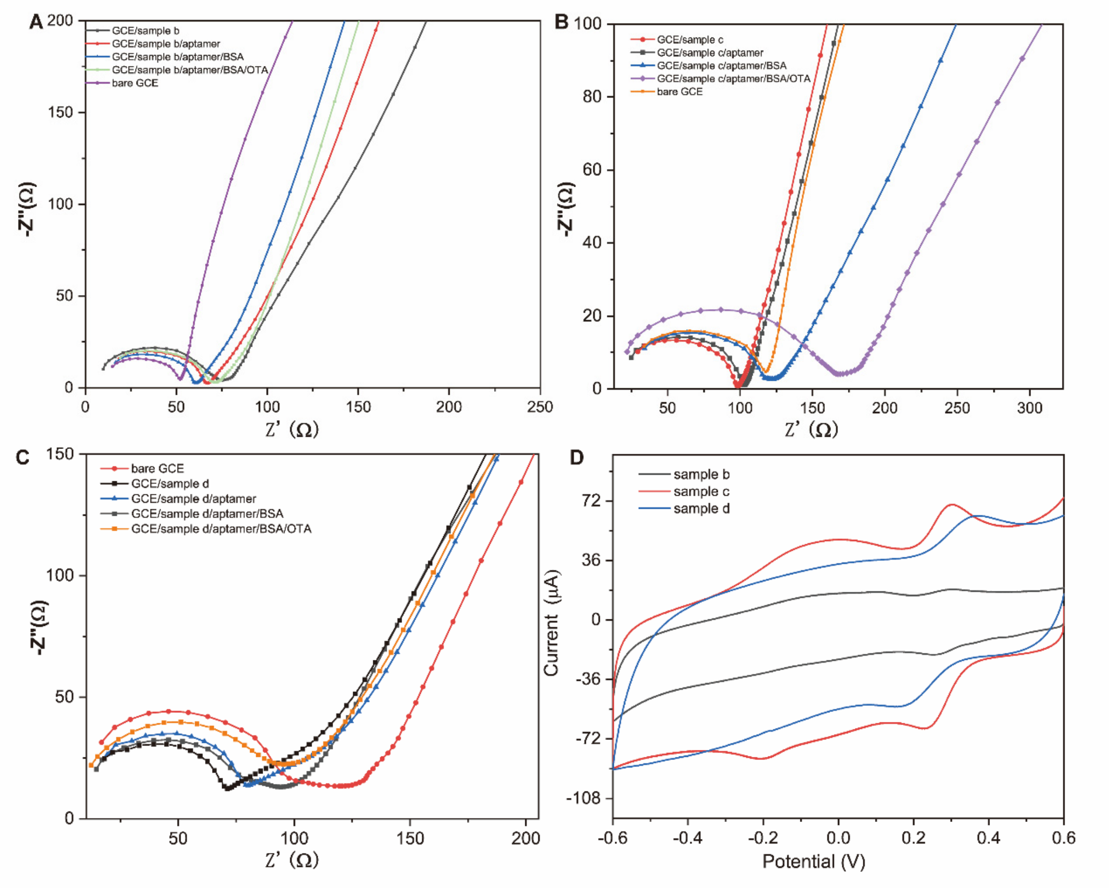

Figure S2 (A-C) The Nyquist plots of GCE after every step of fabrication of nanomaterial (b, c, d), aptamer, BSA, and OTA; (D) CV curves of sample b, c, and d fabricated GCE

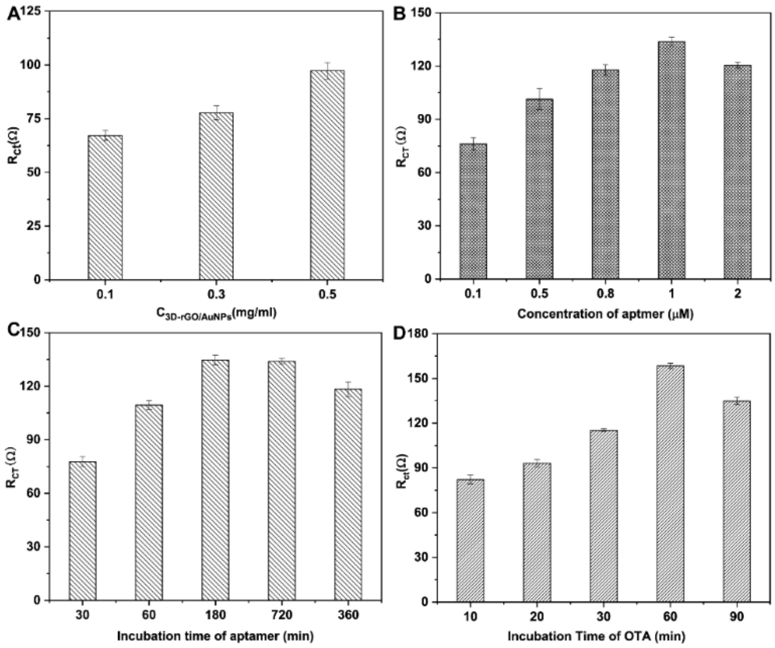

Figure S3 Optimization of (A) concentrations of 3D-rGO/AuNPs, (B) concentrations of aptamer, (C) incubation time of aptamer, (D) incubation time of OTA with aptamer.

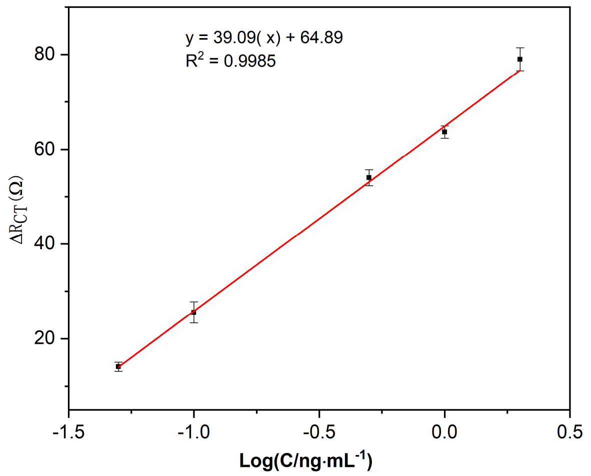

Figure S4 Calibration curve of detection of OTA in red wine based on ΔR_{CT} vs. log (OTA concentration)
